# Updating of pelvimetry standards in modern obstetrics

**DOI:** 10.1038/s41598-024-53603-1

**Published:** 2024-02-06

**Authors:** Caroline Tresch, Marine Lallemant, Camille Nallet, Yvonne Offringa, Rajeev Ramanah, Paul Guerby, Nicolas Mottet

**Affiliations:** 1 Department of Obstetrics and Gynecology, Besancon University Medical Centre, University of Franche-Comté, Besancon, France; 2 Department of Obstetrics and Gynecology, Paule de Viguier University Medical Centre, Toulouse III University, Toulouse, France

**Keywords:** Musculoskeletal system, Anatomy

## Abstract

Clinical value of pelvimetry in modern obstetrics practices has never been established and normal values are set since the middle of the twentieth century. The aim of this study was to describe current dimensions of pelvis in a female French Caucasian population. A retrospective, bi-centric observational study was conducted from August 2013 to August 2019 in two French departments of Obstetrics. We included all Caucasian women who had a computed tomography pelvimetry during pregnancy. The primary outcome was the values of the obstetric transverse diameter, obstetric conjugate diameter and bispinous diameter. Five hundred and fifty-one CT pelvimetries were analyzed. The median Obstetric Transverse Diameter (OTD) was 12.41 cm and the 3rd percentile was 11 cm. The median Obstetric Conjugate Diameter (OCD) was 12.2 cm and the 3rd percentile was 10.5 cm. The median Bispinous Diameter (BSD) in our data collection was 10.9 cm and the 3rd percentile was 9.3 cm. A significant correlation coefficient between women’s height and OTD, OCD and BSD was found. In our study, the OCD and the BSD have not evolved since the middle of the twentieth century. The obstetric transverse diameter was smaller than the standard currently used.

## Introduction

X-ray pelvimetry was introduced in the 1940s by obstetricians to predict the success of a vaginal delivery in cases of suspected cephalopelvic disproportion or breech presentation^1^. Normal values have been established since the middle of the twentieth century. These measures are not consensual. Modern obstetrics textbooks are based on these ancient pelvic measurements of women of European origin, regardless of their ethnic variety^2^. Few teams have published their reference values for judging pelvimetry’s normality. National college of French obstetrician-gynecologists (CNGOF), in its recommendations for clinical practice in cases of breech presentation, notes that the PREMODA study referred to the following values: an Obstetric Transverse Diameter (OTD) ≥ 12 cm, an Obstetric Conjugate Diameter (OCD) ≥ 10.5 cm, a Bispinous Diameter (BSD) ≥ 10 cm^3^. When analyzing the literature about pelvic measurements, it is important to understand which parameter is considered to define a normal value: is it the mean value of the distribution of the 3rd percentile? Actually, the 3rd percentile is used by most authors to define normal value of pelvic diameters. According to Raia-Barjat et al*.*, Neolithic pelvic’s dimensions of the were approximately the same as those of the current basin, but they differ for certain dimensions like the Obstetric Transverse Diameter which was smaller (118 mm vs 125 mm, p = 0.02)^4^. Since the beginning of the twentieth century, the average height of Europeans has increased by one centimeter per decade^5^. The hypothesis of our study is that pelvic measurements have changed over time. 

The baseline pelvic parameters currently used for the interpretation of radiological pelvimetries may no longer reflect the pelvic measurements of French Caucasian parturients. The primary objective of this study was to describe the current pelvic dimensions of Caucasian patients in France. The secondary objective was to evaluate the correlation between height and different pelvic diameters.

## Materials and methods

A retrospective, bi-centric observational study was conducted from August 2013 to August 2019 in the obstetrics departments of the university medical centres in Besançon and Toulouse (France). We included all women who underwent a computed tomography (CT) pelvimetry during pregnancy for any causes. Pelvimetries were identified by clinical coding process. Non-Caucasian women were excluded (when creating the patient's obstetric file, geographic’s origin was requested in consultation). Women with a history of pelvic fracture or constitutional bone disease were excluded.

**Figure 1:**
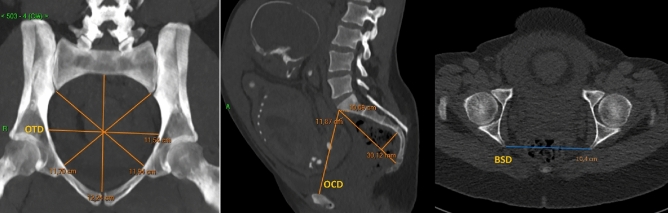
Main pelvic diameters on a CT pelvimetry. *OTD* Obstetric transverse diameter, *OCD* Obstetric conjugate diameter, *BSD* Bispinous Diameter, *CM* centimeters.

Pelvimetries were performed by a 64-channel CT scanner, in a supine position. CT-pelvimetry protocol consisted in a scout-view acquired with a current of 60 mA/s and a tube voltage of 120 kV, and a low-dose helical acquisition with the following parameters: peak tube voltage of 100 kV, tube current–time product of 20–25 mA/s, detector collimation of 16 × 1.2 mm, reconstructed in 3 mm sections using the C filter (corresponding to a moderately sharp reconstruction kernel), and iterative reconstruction (SAFIRE) with a pitch of 0.8 (Somatom Sensation^®^, Siemens Healthineers, Erlangen, Germany). The different obstetrical diameters of the pelvis were measured by multiplanar reconstruction using an image communication and archiving system (Carestream^®^, Carestream Health, Rochester, NY, USA), by radiologists from the University Medical Centres of Besançon and Toulouse. On the axial oblique view through the pelvic inlet and on the mid-sagittal plane, the recorded measurements were as follows OTD, OCD and BSD (Fig. 1). The acquisition included only the pelvis. The OTD bisected the true conjugate (anatomical antero-posterior diameter, from the tip of the sacral promontory to the upper border of the symphysis pubis). The OCD joined the edge of the promontory to the posterior part of the pubis. The BSD connected the two sciatic spines (Fig. 1). Women’s height was determined by self-reporting in centimeters.

The primary outcome was the values of the OTD, the OCD and the BSD. The secondary outcome measurement was women’s height.

Data were extracted from women’s electronic and paper medical records. The criteria collected were maternal and CT-pelvimetric characteristics. Maternal caracteristics we analysed were maternal age, weight before pregnancy, height, body mass index (BMI) and primiparity in order to compare to others studies. We also collected the history of scoliosis because this could be a bias. A retrospective analysis showed that patients with degenerative lumbar scoliosis may have a higher pelvic incidence. In this study the sacral slope were lower for patients with degenerative lumbar scoliosis, the scoliosis Cobb’s angle was correlated with pelvic tilt and the thoracic kyphosis was correlated with sacral slope, and pelvic tilt^6^. The history of scoliosis could therefore have an influence on pelvis measurements values or may influence obstetric dynamics.

Useful diameters for acceptance of vaginal breech delivery were studied: OTD, OCD and BSD.

Data were collected anonymously in a secured Excel spreadsheet. Missing data were not replaced. The 3rd, 10th, 50th, 90th and 97th percentile, mean and standard deviation were calculated for each diameters^5^. Pearson’s correlation coefficients were calculated between women’s height and the different pelvic diameters. Diameter distributions were presented by scatterplots and regression lines. The statistical study was carried out using R software version 4.1.2^®^.

This study was an observational trial using anonymized data from medical records. In both centres, women were systematically informed that obstetric and neonatal data could be used for the evaluation of medical practices and were explicitly informed that they could signed an objective form. All participants provided written informed consent. Our study was performed in accordance with relevant guidelines and regulations and in accordance with the Declaration of Helsinki. The study protocol was approved by the Institutional Review Board of the French College of Obstetricians and Gynecologists (CEROG n°2020-OBS-0406).

## Results

During the study period, 624 women underwent a CT pelvimetry. Thirteen women were excluded because of a history of pelvic fracture and sixty because of a non-Caucasian origin. Finally, 551 CT pelvimetries were analyzed. These pelvimetries were performed at 36–37 weeks of gestation and were indicated for a breech presentation (n = 517/551, 94%) and for a cephalic presentation in case of history of road accident or lameness (n = 34/551, 6%).

**Figure 2:**
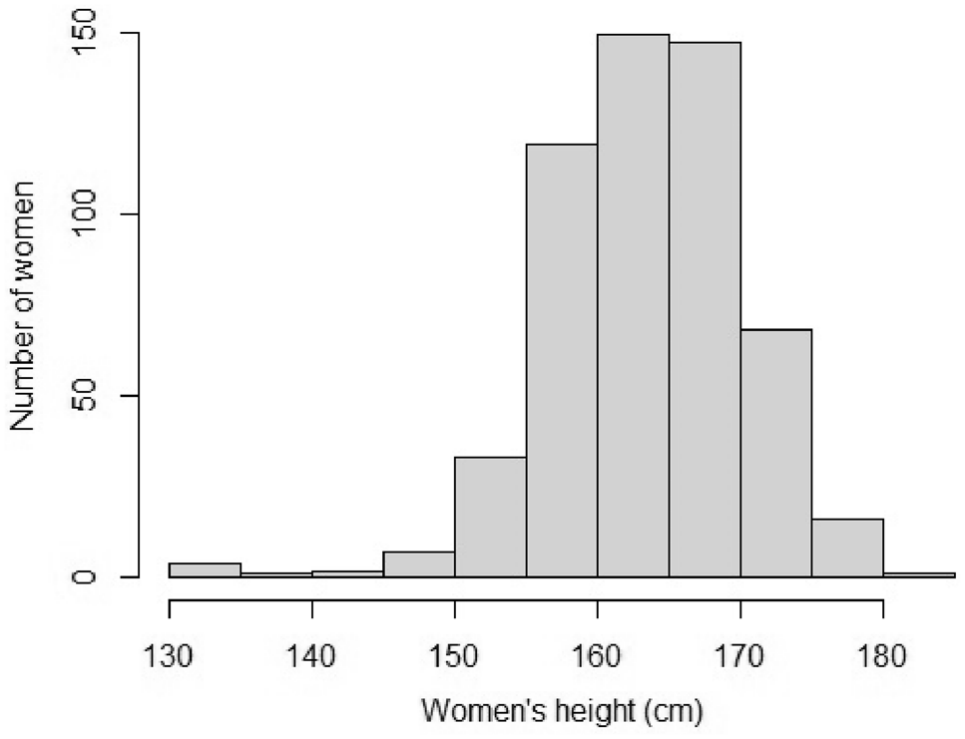
Women’s height distribution. *CM* centimetres.

Maternal characteristics are summarized in Table 1. The women’s height had a normal distribution (Fig. 2). CT pelvimetric measurements are presented in Table 2.

**Table 1:** Maternal characteristics of Caucasian women (n = 551).

Maternal age (years)	29.8 ± 5.0
Weight before pregnancy (kg)	63.3 ± 13.7
Height (cm)	164 ± 9.7
BMI before pregnancy (kg/m^2^)	23.0 ± 4.5
Primiparity	374 (68)
History of scoliosis	3 (0.5)

Values are presented as mean ± deviation standard or number of cases (percentage).

*BMI* body mass index, *kg* kilograms, *cm* centimetres.

**Table 2:** CT-pelvimetric results.

	3rd p	10th p	50th p	90th p	97th p	Standard deviation	Mean
OTD	11.00	11.50	12.41	13.50	14.00	0.77	12.47
OCD	10.50	11.00	12.20	13.60	14.25	1.01	12.27
BSD	9.30	9.70	10.90	12.24	13.26	1.04	10.97

Measurements are in centimeters.

*P* percentile, *OTD* obstetric transverse diameter, *OCD* obstetric conjugate diameter, *BSD* bispinous diameter.

**Figure 3:**
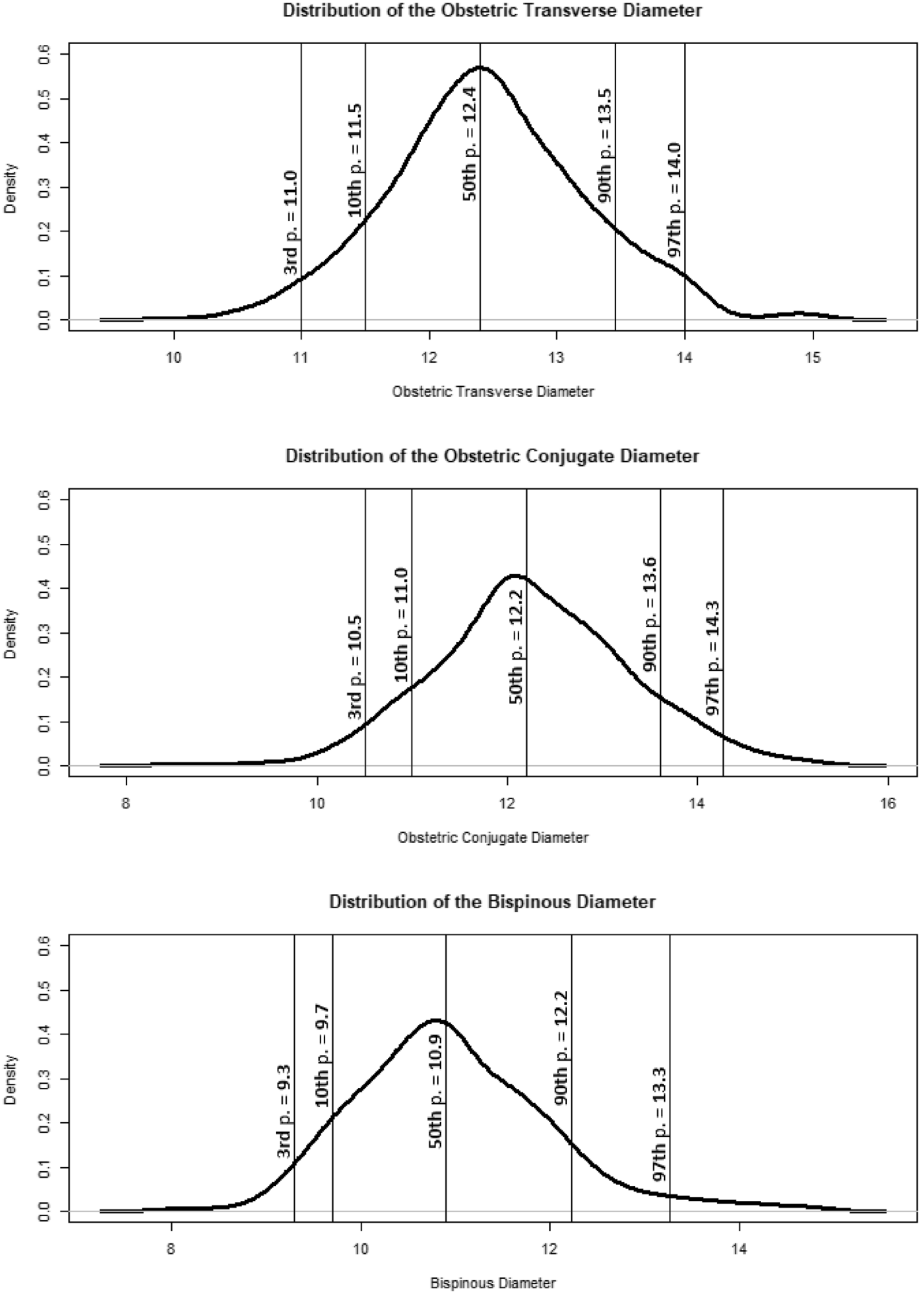
Obstetric transverse diameter, conjugate diameter and bispinous diameter measurements distribution in centimeters. *p* percentile.

Measurements of the OTD, the OCD and the BSD followed a Gaussian distribution (Fig. 3). One hundred and thirteen patients had an obstetric transverse diameter between 11 and 11.9 cm (20.5%).

**Figure 4:**
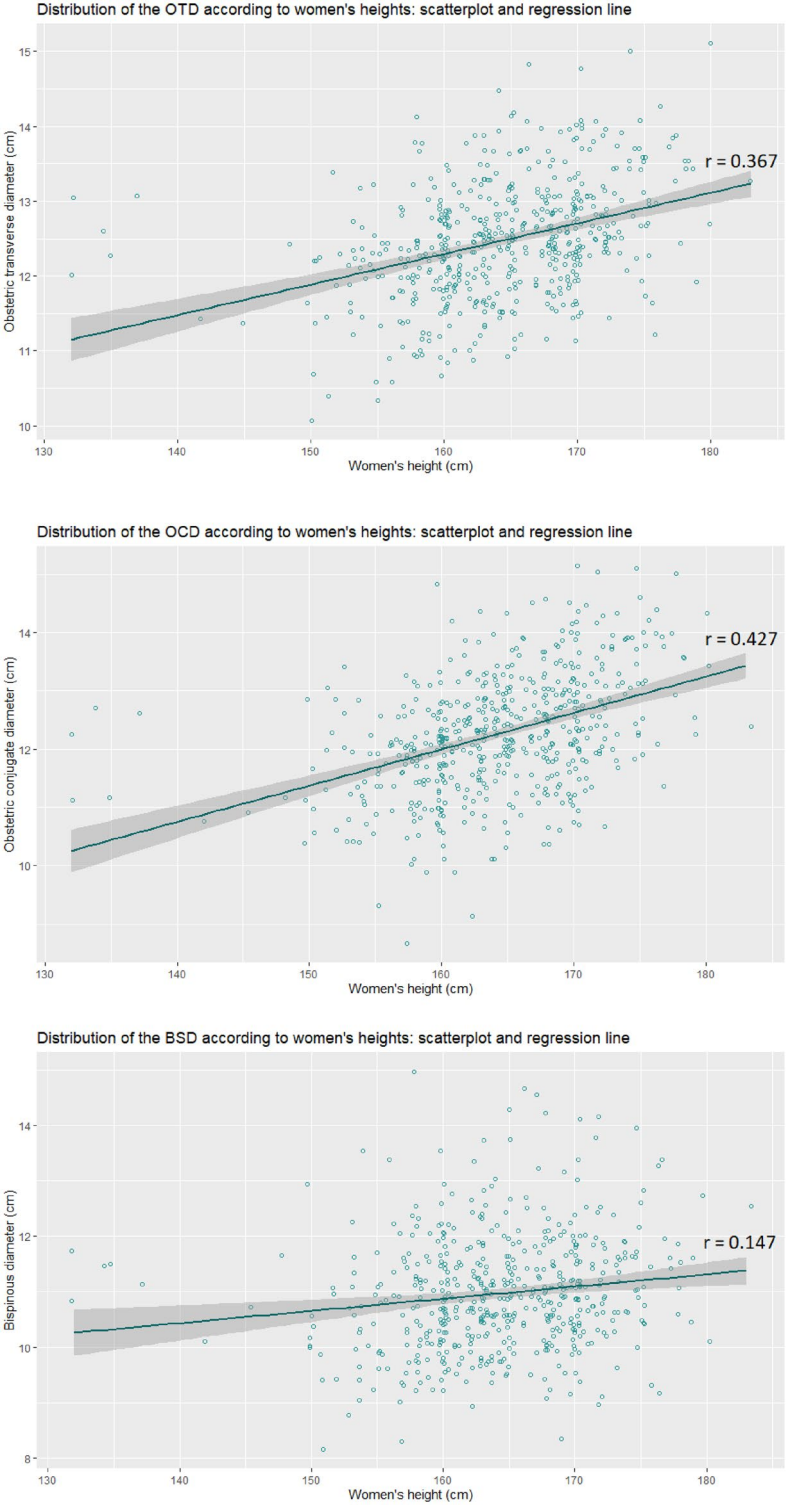
Distribution of obstetric transverse diameter, obstetric conjugate diameter and bispinous diameter according size’s women scatterplot and regression line, *cm* centimeters.

A statistically significant correlation coefficient between height and obstetric transverse diameter was found: r = 0.3643 (95% confidence interval [0.2892; 0.4349], p < 0.01), between height and obstetric conjugate diameter: r = 0.3924 (95% confidence interval [0.319; 0.4611], p < 0.01) and between height and bispinous diameter: r = 0.1408 (95% confidence interval [0.0573; 0.2223], p < 0.01). Trend lines of OTD, OCD and BSD distribution as a function of height were drawn (Fig. 4).

## Discussion

The current standards of interpreting of CT-pelvimetries no longer fully reflect the pelvic measurements of women delivering in France. Through our series of 551 radiological pelvimetries, we were able to study the distribution of pelvic measurements in two French Medical Centres over the last 6 years. The relevance of pelvimetry is debated because there is no consensus on normal pelvic values. Currently, the only unanimous indication of pelvimetry is breech presentation and there is no argument to recommend pelvimetry in the event of delivery before 37 weeks of gestation^7^. In addition, pelvic measurements may depend on ethnic factors and patient heights^2,5^. Betti and Manica’s study showed that the relationships between the different pelvic measures were different depending on ethnicity^2^. For example, the ratio of anteroposterior diameter of the inlet (from the sacral promontory to the dorsomedial superior pubis) to the mediolateral diameter of the inlet (maximum distance between the linea terminalis) was higher in Asian, Sub-saharian and African populations compared to European pelvis measurements^2^.

Maternal characteristics of our study were similar to Michel et al*.*^8^, Keller et al*.*^9^ and Lenhard et al*.*^10^ studies in terms of age, height, weight and BMI. Regarding height, this corresponds to the French Women average^11^. Women’s number with a history of scoliosis was negligible.

According to published data, the radiological pelvimetry values for accepting a trial of vaginal breech delivery in French labours wards are: an OTD ≥ 12 cm, an OCD ≥ 10.5 cm, a BSD ≥ 10 cm^12^. In our study, the OTD’s 10th percentile was 11.5 cm, the 3th percentile was 11 cm and 20.5% of women had an OTD between 11 and 11.9 cm. Thus, the limit of 12 cm used in the Premoda study appeared restrictive and the choice of the 3rd percentile (11 cm) or the 10th percentile (11.5 cm) would be more relevant to define the threshold value. The distribution of these values observed in our study could also be explained by the non-Caucasian migration flows that occurred during the second half of the twentieth century. Even if our population was Caucasian, it was difficult to know the exact origins of the patients, because France is a mixed country. Pelvic width was smaller in African and South-East Asian populations^2,5^. Indeed, pelvic width and body mass index would be higher in populations living in cold climates such as France due to thermal adaptation^5^. For example, Handa et al. compared pelvis measurements of white American women to African-American women and found a statistically significant difference in median transverse diameter (12.6 ± 0.7 vs 11.8 ± 0.7, p < 0.001)^13^. According to Nicholson and Sanderman Allen^14^, Michel et al*.*^8^, Keller et al*.*^9^ and Lenhard et al*.*^10^ studies, the mean OTD would vary between 12.9 and 13.1 cm, and the 3rd percentile between 11.1 and 11.6 cm (Table 3). Demographic characteristics in our study were similar to pelvimetry cohorts of these authors.

**Table 3:** Distribution of OTD, OCD and BSD values (in centimeters) according to different authors.

	Authors	3rd p	Mean
OTD	Nicholson and Sandeman Allen^14^	11.6	13.1
	Michel et al.^8^	11.5	12.9
	Keller et al.^9^	11.2	13.0
	Lenhard et al.^10^	11.1	12.9
	Our study	11.0	12.5
OCD	Nicholson and Sandeman Allen^14^	10.2	12.1
	Michel et al.^8^	10.6	12.4
	Keller et al.^9^	10.5	12.2
	Lenhard et al.^10^	10.2	12.0
	Our study	10.5	12.3
BSD	Nicholson and Sandeman Allen^14^	8.9	10.5
	Michel et al.^8^	9.6	11.0
	Keller et al.^9^	9.7	11.2
	Lenhard et al.^10^	9.5	10.9
	Our study	9.3	11.0

*P* percentile, *OTD* obstetric transverse diameter, *OCD* obstetric conjugate diameter, *BSD* bispinous diameter.

According to Schaal et al*.*, the value currently considered normal for OCD is 10.5 cm and a pelvis would be considered subnormal between 8.5 and 10.5 cm^15^. The standard value of 10.5 cm currently used corresponded to the 3rd percentile of our data. Our results were in agreement with Nicholson and Sandeman Allen^14^, Michel et al*.*^8^, Keller et al*.*^9^ and Lenhard et al*.*^10^ studies in which the mean OCD was between 12 and 12.4 cm and the 3rd percentile was between 10.2 and 10.6 cm (Table 3). Conversely to the OTD, the OCD would not be correlated with ethnicity according to Gabriel^5^ and Handa et al*.* who did not find a statistically significant difference between White and African-American women for the OCD (12.3 cm ± 1.1 vs 12.1 cm ± 1.0, p = 0.25)^13^.

According to Schaal et al*.*, the BSD was considered normal if it was higher than or equal to 9.5 cm^15^. Pelves were considered subnormal between 8 and 9.5 cm^14,15^ and the mean value of the bispinous diameter was 10 cm^15^. For Nicholson and Sandeman Allen^14^, Michel et al.^8^, Keller et al.^9^, Lenhard et al.^10^, the 3rd percentile would be between 8.9 and 9.7 cm and the mean between 10.5 and 11.2 cm (Table 3). In Shirley et al. study, the BSD’s mean of 68 young nulliparous women of South Asian origin was similar (10.9 ± 0.7 cm)^16^ and Handa et al. found no statistically significant difference in BSD between White American women and African-American women (10.5 cm ± 0.8 vs 10.3 cm ± 0.9, p = 0.15)^13^.

Finally, the mean value and the 3rd percentile have not changed concerning the OCD and the BSD and the 3rd percentile is often taken as the threshold value of normality.

Regarding the correlation between height and different pelvic diameters, according to Gabriel and our data, the OTD would be correlated with height^5^. Conversely to the OTD, the OCD would not be correlated with height according to Gabriel^5^. In our study, height had a correlation on OCD (correlation coefficient 0.3924). In Keller et al*.* study, the OCD and the BSD was correlated with height in a group of 743 women^9^. In our study, the correlation between height and bispinous diameter was present, but weak. This information could be taken into account during obstetric decisions for small patients, but it does no call into question the relevance of CT scans.

The main limitation of our study was that it was a retrospective study, which could lead to compendiums bias. Then, pelvis studied are those of pregnant women and we can wonder if the measurements of the pelvis change during pregnancy (which could also be the subject of another study). However, since pelvimetry is of interest during pregnancy, particularly in cases of breech presentation, the clinical implication is more interesting in pregnant women close to term.

Ethnicity could be a bias. In our obstetrics software, there were an item “nationality” and an item “geographic origin”. So the doctor or midwifes directly inquired to the patient during medical consults. But as this is a retrospective study, we are not sure that the question was systematically asked. It is possible that for some patients the staff completed the geographical origin item without asking the question (Africa, Asia, Europe…). It could be also difficult to classify women whose parents came from two different countries. Another limitation was a possible inter-observer variability in the measurements because they were performed by different radiologists. However, results followed a Gaussian distribution, which was consistent with the reliability of measurements.

The main strength of this study was the large cohort of CT-pelvimetries performed in two obstetric referral centres and performed by trained radiologists. It represents the most contemporary update to the field, considering other studies are dated by over a decade. The most interesting result of our study is that 20.5% of women had OTD between 11 and 11.9 cm. We can ask if we can really consider that 20.5% of women have a narrowed pelvis or if current standards are too restrictive.

## Conclusion

In our study, the obstetric conjugate diameter, which would be a species characteristic has not evolved. The obstetric transverse diameters were smaller than the currently used standard. Bispinous diameters has not evolved. It would be interesting to evaluate the obstetrical prognosis according to these new values: a 3rd percentile at 11 cm for OTD, at 10.5 for OCD, at 9.30 for BSD. For example, the limit of 12 cm used in the Premoda study to define normal OTD appeared restrictive and the choice of the 3rd percentile (11 cm) or the 10th percentile (11.5 cm) would be more relevant to define the threshold value.

## Data Availability

The datasets generated during and/or analysed during the current study are available from the corresponding author on reasonable request.

## References

[1] Rozenberg P (2007). Is there a role for X-ray pelvimetry in the twenty-first century?. Gynecol. Obstet. Fertil..

[2] Betti L, Manica A (2018). Human variation in the shape of the birth canal is significant and geographically structured. Proc. R. Soc. B Biol. Sci..

[3] National college of French obstetrician-gynecologists (CNGOF). Recommendation for clinical practice: breech presentation (2020).

[4] Raia-Barjat T, Tardieu A-S, Amouzougan A, Trombert B, Chauleur C, Varlet M-N (2011). Analyse anthropométrique du bassin obstétrical datant du Néolithique: Conséquences obstétricales Étude préliminaire. Gynécologie Obstétrique Fertil..

[5] Gabriel, R. Harika, G. Bonneau S. Bassin obstétrical : anatomie, étude clinique et radiologie. EMC - ObstétriqueGynécoloe 11 (2016).

[6] Han F, Weishi L, Zhuoran S, Qingwei M, Zhongqiang C (2017). Sagittal plane analysis of the spine and pelvis in degenerative lumbar scoliosis. J. Orthop. Surg. Hong Kong.

[7] Azria É (2020). Breech presentation: CNGOF guidelines for clinical practice—Case selection for trial of labour. Gynecol. Obstet. Fertil. Senol.

[8] Michel SCA, Rake A, Treiber K, Seifert B, Chaoui R, Huch R (2002). MR obstetric pelvimetry: Effect of birthing position on pelvic bony dimensions. Am. J. Roentgenol..

[9] Keller TM, Rake A, Michel SCA, Seifert B, Efe G, Treiber K (2003). Obstetric MR pelvimetry: Reference values and evaluation of inter- and intraobserver error and intraindividual variability. Radiology.

[10] Lenhard MS, Johnson TRC, Weckbach S, Nikolaou K, Friese K, Hasbargen U (2010). Pelvimetry revisited: Analyzing cephalopelvic disproportion. Eur. J. Radiol..

[11] Saint Pol De T. Body and social belonging: corpulence in Europe (Corps et appartenance sociale: la corpulence en Europe). Natl Inst Stat Econ Stud https://www.insee.fr/fr/statistiques/1371911?sommaire=1372045. (2006).

[12] Mattuizzi A (2020). Breech presentation: CNGOF guidelines for clinical practice—epidemiology, risk factors and complications. Gynecol. Obstet. Fertil. Senol..

[13] Handa VL, Lockhart ME, Fielding JR, Bradley CS, Brubakery L, Cundiffy GW (2008). Racial differences in pelvic anatomy by magnetic resonance imaging. Obstet. Gynecol..

[14] Nicholson C, Allen HS (1946). Variation in the female pelvis. Lancet Lond. Engl..

[15] Schaal JP., Riethmuller D., Maillet R., Uzan M. Dystocies osseuses. Mécanique Tech. Obstétricales. 4ème, Montpellier: Sauramps Medical 433–61 (2012).

[16] Shirley MK, Cole TJ, Arthurs OJ, Clark CA, Wells JCK (2020). Developmental origins of variability in pelvic dimensions: Evidence from nulliparous South Asian women in the United Kingdom. Am. J. Hum. Biol..

